# Determination of diagnostic standards on saturated soil extracts for cut roses grown in greenhouses

**DOI:** 10.1371/journal.pone.0178500

**Published:** 2017-05-25

**Authors:** John Jairo Franco-Hermida, María Fernanda Quintero, Raúl Iskander Cabrera, José Miguel Guzman

**Affiliations:** 1 Technical Department, G.R. Chía S.A., Centro Empresarial Chía, Chía, Colombia; 2 Department of Controlled Production Systems, Faculty of Agronomy, San Luis Potosí Autonomous University, San Luis Potosí, México; 3 Department of Plant Biology, Rutgers University, Bridgeton, NJ, United States of America; 4 Department of Agronomy, cei A^3^ CIEMBITAL Almeria University, Almería, Spain; Laval University, CANADA

## Abstract

This work comprises the theoretical determination and validation of diagnostic standards for the analysis of saturated soil extracts for cut rose flower crops (*Rosa* spp.) growing in the Bogota Plateau, Colombia. The data included 684 plant tissue analyses and 684 corresponding analyses of saturated soil extracts, all collected between January 2009 and June 2013. The tissue and soil samples were selected from 13 rose farms, and from cultivars grafted on the 'Natal Briar' rootstock. These concurrent samples of soil and plant tissues represented 251 production units (locations) of approximately 10,000 m^2^ distributed across the study area. The standards were conceived as a tool to improve the nutritional balance in the leaf tissue of rose plants and thereby define the norms for expressing optimum productive potential relative to nutritional conditions in the soil. To this end, previously determined diagnostic standard for rose leaf tissues were employed to obtain rates of foliar nutritional balance at each analyzed location and as criteria for determining the diagnostic norms for saturated soil extracts. Implementing this methodology to foliar analysis, showed a higher significant correlation for diagnostic indices. A similar behavior was observed in saturated soil extracts analysis, becoming a powerful tool for integrated nutritional diagnosis. Leaf analyses determine the most limiting nutrients for high yield and analyses of saturated soil extracts facilitate the possibility of correcting the fertigation formulations applied to soils or substrates. Recommendations are proposed to improve the balance in soil-plant system with which the possibility of yield increase becomes more probable. The main recommendations to increase and improve rose crop flower yields would be: continuously check pH values of SSE, reduce the amounts of P, Fe, Zn and Cu in fertigation solutions and carefully analyze the situation of Mn in the soil-plant system.

## Introduction

The maintenance of a vigorous, aesthetics and sustained year-round productivity in Colombia’s greenhouse roses is associated with high nutrient requirements. In practice, applications of fertilizer significantly exceed demand by the crop. The main problems of this excessive fertigation in rose crops are associated with the elements nitrogen (N), phosphorus (P), magnesium (Mg), manganese (Mn) and boron (B), which had very significant effects on the nutritional balance and productivity [1].

For determining the nutritional status of crops, the greenhouse flower industry uses the analysis of extractable elements from soils or substrates [2]. Analysis of saturated soil paste extracts (SSE) provide more dynamic information about the availability of nutrients than classical soil test methods [3,4]. However, these results are not practical for fertigation management planning in greenhouse crops. The concentrations of elements applied with fertigation scarcely modify the magnitude of elemental concentration reported in this type of analysis. This fact increases the difficulty of assessing the influence of fertigation on soils and subsequently on the plants.

The commonly used laboratory method for extracting soil solution is the saturated soil paste extract [5]. For protected crops subjected to fertigation management conditions, it has been asserted that the most appropriate analysis of the substrate solution is to use distilled water as the extracting agent, preferable over the use of fertigation solution [6].

In addition to the choice of the extracting agent for SSE, equally important is the test results interpretation method. Currently, the most commonly used methods are the Critical and/or Reference Levels of each individual element [7]. These methods ignore the relationships between the concentrations of different elements, which largely determine their availability to plants [8–11]. The Diagnosis and Recommendation Integrated System (DRIS) and Compositional Nutrient Diagnosis (CND) methodologies include the concept that the relationships between the elements facilitate improved diagnostics rather than the isolated concentration values of each one [7,12,13]. These methodologies have proven to be a better tool for the interpretation of the analytical information, than the individual concentrations of the elements [7,12–15]. An added benefit from the correction and optimization of fertilization practices using these integrative nutrient diagnostic procedures is the potential for reductions in both fertilizer use (cost savings) and its environmental impacts (leaching and runoff losses).

After Parent and Dafir [16], many authors have already qualified CND as a more efficient method than DRIS to determine the nutritional status of crops [1,17–22], including those growing under hydroponics [23]. This work not only attempts to corroborate these observations in SSE, (correlated with its corresponding foliar tissue analyses), but provide useful information for Colombian producers and their crop advisors, providing decision-making tools to equilibrate the soil-plant system at high-yield levels. From a practical point of view, it is suitable to offer more than one option to introduce users to these integrated diagnostic tools.

The main objective of this work is to obtain integrated diagnostic norms based on SSE analysis, enabling the correction and optimization of fertigation practices in cut rose crops in the Bogotá Plateau flower growing region of Colombia. This objective was addressed in two consecutive steps: (1) Determine the DRIS and CND norms for SSE analysis in cut rose cultivation and (2) validate their functionality at the theoretical level.

## Materials and methods

The study area includes 13 farms distributed throughout the Bogotá Plateau of Colombia, one of the largest cut-flower growing regions in the world. In this region the three main soil types are Andisols, Inceptisols and Entisols, among which there are Hapludands, Melanudands, Dystrustepts, Haplustepts, Endoaquepts and Ustorthents [24]. The predominant textures are clay-loam and clay. These soils, however, have had significant changes in their physicochemical properties because of many years of intensive fertigation under greenhouse cultivation. Average values of soil chemical analysis results for this study region are presented in Table 1.

**Table 1:** Average values and confidence interval (n = 9555 and p ≤ 0,05) of the chemical characteristics of soils dedicated to floriculture on the Bogotá Plateau, Colombia. Values obtained from the database of the G.R. Chia SA Soil Laboratory.

	Average	Confidence interval
pH	6,4	6,39–6,41
CE	2,1	2,11–2,16
O.M. (%)	8,5	8,46–8,63
CEC	20,3	19,9–20,6
N-NH4	31,8	31,6–32
N_NO3	157,6	156–160
pH	206,7	204–209
S	257,4	253–261
K	982,7	971–995
Ca	4468,1	4434–4502
Mg	837,3	829–845
Na	280,4	275–285
Fe	137,5	136–139
Mn	14,9	14,7–15,2
Cu	7,4	7,25–7,63
Zn	17,6	17,2–17,9
B	2,8	2,78–2,85
Ca (% sat.)	69,9	69,4–70,4
Mg (% sat.)	21,4	21,2–21,6
K (% sat.)	7,9	7,79–7,97
Na (% sat.)	3,9	3,79–3,92
Ca/Mg	3,6	3,59–3,65
Mg/K	3,4	3,35–3,48
Ca/K	11,3	11,1–11,5
(Ca + Mg)/K	14,7	14,4–15

Methods (IGAC 1990): pH (1:1); EC saturated extract; OM (Organic Material, Walkley-Black); CEC (Cationic Exchange Capacity), K, Ca, Mg, Na ammonium acetate pH 7; N-NH_4_ and N-NO_3_ extraction with potassium chloride; P Olsen; Fe, Mn, Cu, Zn extraction with DTPA; Boron hot water extraction.

From January 2009 to June 2013, soil and plant tissue samples were collected from 251 plots within this study area. All necessary permits were obtained from its owner, G.R. Chia SA, which complied with all relevant regulations. Rose plots under plastic greenhouse were cultivated employing drip fertigation methods, with different cultivars of roses grafted on the rootstock *Rosa* × 'Natal Briar'. The nutrient solution formulations used in these crops had the following composition (in mg·L^-1^): 140 to 180 nitrogen (N), 30 to 40 phosphorus (P), 150 to 200 potassium (K), 100 to 150 Ca, 40 to 70 Mg, 0.5 to 1.0 manganese (Mn), 0.5 to 2.0 iron (Fe), 0.1 to 0.5 copper (Cu), 0.2 to 0.6 zinc (Zn), and 0.5 to 1.0 boron (B) [1].

Soil cores were obtained from the top 30cm (rootzone) with a borehole of 3cm in diameter, employing a zigzag pattern and covering each entire plot. Three subsamples were taken from each plot, mixed, homogenized and labeled by date and plot, constituting a soil sample. These samples were taken to the laboratory where soil solution extractions were performed [5]. Throughout the study, 684 samplings were performed.

Leaf tissues were collected following a similar zigzag triplicate-subsampling pattern from plants located in the same plots where soil samples were taken.

Samples of both SSE and foliar tissues were analyzed at the soil and plant laboratory of GR. Chia S.A. employing the methodologies proposed by IGAC [2] for soil, and Franco et al. ([1]) for leaf tissues. The concentration of mineral elements was expressed in mg·L^-1^ for SSE and in ppm for foliar tissues.

A "Source" database was created using analytical (SSE and leaf) data obtained from these 251 plots between January 2009 and March 2011 (samples collected on different dates). The DRIS and CND norms for both SSE and foliar tissues were obtained from this database source.

A "Validation" database was also created with analytical (SSE and plant tissues) data collected from March 2011 until June 2013. This database was used to perform the theoretical validation of the previously obtained diagnostic norms.

### Determination of DRIS norms

As previously indicated, the "Source" database was used for the determination of the DRIS norms. The concentrations of Fe, Mn, Cu, Zn and B were multiplied by 100 to obtain manageable figures and avoid excessive variances [25]. The functions for the elements and the DRIS indices were determined according to the methodology of Beaufils [26,27].

With the aim of establishing a direct relationship between the balance of elements in soil and the nutritional balance in plants, we used the foliar DRIS Nutritional Balance Index (NBI*f*) as a criterion to separate populations, instead of using flower crop yields. To establish this relationship, the NBI*f* values were calculated (from N, P, K, Ca, Mg, S, Na, Fe, Cu, Mn, Zn, B foliar concentrations) according to the Beaufils method [13], and cross referenced with the data for each of the corresponding SSE as a reference value for the separation of populations. This was done as rose crops with NBI*f* values >60 have the nutritional conditions to be considered within the high productivity population [1]. In this way, the two SSE data populations were separated: one with high- and another with a low NBI*f*.

Considering the mentioned variation, the DRIS norms were calculated following the classic method of Beaufils [14,15,28,29], also, applied by Franco et. al. [1] in the determination of foliar DRIS norms for cut roses.

### Determination of CND norms

In order to calculate the CND norms, or centered proportions, the methodology proposed by Parent [12,30] was applied to the analysis of each SSE. CND as used here consists of centered log ratios. The log expression is reflective and symmetrical, i.e. log(X/Y) = log(Y/X), and nutrient interactions are accounted for using a geometric mean. New balance concepts are being elaborated [12] using isometric log ratios (rather than centered log ratios) to diagnose plant nutrients. According to Franco at al. [1], critical CNDr^2^ foliar values <7.4 indicate a high probability that a sample has a high nutritional balance and a correspondingly high flower yield. Therefore, to separate populations and to select standards, any SSE sample that had an associated CNDr2 foliar index (CND*f*) <7.4 was considered within a high nutritional balance population.

### Theoretical validation of the DRIS and CND norms

The validation of the norms was based on the assumption that there must be significant correlation between indices of nutritional balance in plant tissue and nutritional balance in the SSE indices [14,31]. This hypothesis suggests that the nutritional balance in the soil determines to some extent the nutritional balance in plant tissues, and simultaneously allows differentiation of samples in which nutritional imbalances in the SSE affect the nutritional balance in plant tissues.

The “Validation” database was used to validate the DRIS and CND norms for nutritional diagnosis. For each methodology, the indices of nutritional balance in the soil (NBI*s*) were compared using a correlation analysis with the respective index of nutritional balance in the foliar tissue (NBI*f*). If the correlation is significant it means that standards for soil may be helpful to determine nutritional balance in the plants.

## Results and discussion

The “Source” database comprises analytical values obtained from soil samples taken from a large number of rose crops (grafted on *Rosa*. x 'Natal Briar') over a period of five years. The samples were taken throughout the crop’s entire cycle at different locations, including a range of soil types and under differing weather conditions to reflect the variations typical to the change in seasons. The overall level of productivity of each crop was related to each of the analytical data obtained from their SSE. The inclusion of a wide range of sampling data is the established method to obtain the diagnostic norms to be used as fertigation recommendations criteria [15,29,32,33].

### DRIS norms

#### Separation of populations

Basic statistics from the SSEs of high and low NBIf populations are presented on S1 Table. F-test [15,29] and t-test [28] were applied to EC, pH and each elemental concentration [E], to compare populations. The variance in the high-yield population (α = 0.05) is significantly lower for 10 of the 14 analysed elements compared to that of the low-yield population. This observation is consistent with the principles of the DRIS system [27] and is an accurate indicator of the reliability of the norms [14,29].

Variance and mean values (S1 Table) show significant differences in EC between the two populations. This aspect is relevant in plant nutritional diagnosis because it indicates that not only the nutritional balance is considered, but also the influence of the total concentration of salts. [8,11,34–40]. In this sense, crops may have nutritional disorders caused by salinity such as reductions in nitrate and phosphate absorption and metabolic disorders related to Ca^2+^, NH_4_^+^, K^+^ and Mg^2+^. In addition, under high salinity conditions (high EC) the activity of the elements in the nutrient solution is reduced and thus their availability to plants [8–11].

Conversely, pH did not present significant differences between populations (S1 Table), and according to several authors [41–44], the observed average pH values can be considered normal for growth and productivity of cut rose crops.

The statistical values of phosphorous (P) show significant differences between the two populations, both in variance (*F*-test) and in means (*t*-test); in addition, the coefficient of variation for this element is very high, even in the population of high NBI*f*. The complex interactions that occur in the Bogotá plateau soils between P and the amount of organic matter and ashes present in them (IGAC 2000), hinder the management of phosphorous nutrition in the region. This manifest itself through significant accumulations of P in soils (**Table 1**) because of excessive application of P fertilizer. (S1 Table) shows a reduced mean P value in the high NBI*f* population compared to the low NBI*f*, possibly indicative that intense P fertilization may have negatively affected its nutritional balance.

The population of high NBI*f* presents a higher value and a lower variance for Mn than that of low NBI*f*. This is an important aspect given the low levels of manganese found (**Table 1**) in the soils of this rose growing region [24]. This leads to the conclusion that the addition of manganese in fertigation improves the nutritional balance of plants and thus probably the potential flower productivity of rose crops.

Sodium and Cl values of the SSE (S1 Table) did not show significant differences in means or variances for either of the two populations. The critical values above which rose crops manifest physiological disorders, are 4 mM of Na and Cl (92 mg L^-1^ Na^+^ and 142 mg L^-1^ Cl^-^) [36]. This is true for the average values of Cl^-^ observed in both rose populations, but with an elevated coefficient of variation (62% and 66% for low and high NBI*f* populations respectively), that can generate temporal conditions of toxicity in some individual soils. However, the average Na^+^ concentration values were high in both populations (S1 Table) as compared to the acceptable values listed for rose plants [34,36]. The mean values of exchangeable Na percentages (**Table 1**) were below 15% so these soils cannot be considered sodic [5]. This is mainly due to exchangeable bases of CEC are mainly occupied by Ca^2+^, Mg^2+^ and K^+^ (Table 1). The relative concentrations of Cl^-^ and Na^+^ in soils affects, to some extent, the nutritional balance of plants [8,34]. For this reason, the relationship of these two elements with the other elements is important and justifies their use within the DRIS norms for SSE. Nevertheless, their behavior is very similar in populations of low and high NBI*f*.

Boron also shows no significant differences in the *F*-test and *t*-test. Boron is an element of particular importance in the management of rose crops, and their deficiency or excess can produce significant and similar metabolic disorders [34,42,45,46]. Moreover, B is strongly linked to Ca metabolism in plants [10,45,47]. Calcium is directly related to the development of rose crops, and largely determines their flower yield and postharvest quality [47–50].

#### Ratio significance and DRIS norms

S2 Table shows DRIS norms, coefficients of variation (CV), F- and t-tests for elemental ratios obtained in the high yield population (NBIf < 60).

When comparing the norms (S2 Table) with the relations of the population of low NBI*f*, shown that 86 from 91 norms have a significantly lower variance, 31 of which also have significant differences in the mean. A large number of norms with differences in the variance or in the mean with respect to the low yield population suggests that the norms are reliable [28], and may indicate differences in the nutritional balance of the plants arising from elemental imbalances in the soil.

When there is a very low variance for a norm in the population of high NBI*f*, and additionally the CV of the norm is relatively low, the crop is more susceptible to a small change in nutritional balance [51].

The DRIS norms presented for SSE (S2 Table) show significance in the *F*-test values >2% and <50% of CV for the ratios K/N-NO_3_, Ca/N-NO_3_, Mg/N-NO_3_, N-NO_3_/S, 100B/N-NO_3_, Mg/Ca, Ca/S, Mg/S, 100Cu/S, 100Zn/S, 100B/S, 100Cu/100B. This suggests that small changes in the concentrations of these elements in soils have a marked impact on nutritional balance in the rose plants and can therefore cause changes in flower yields. These data are consistent with results obtained by Franco et al. ([1]) for cut rose crops in the Bogotá plateau. Nitrogen, S, Mg and K are the critical elements for nutritional balance in the plant tissues and are decisive for crop yields.

#### Theoretical validation of DRIS norms

Correlations between the nutritional balance indexes in plant tissue (NBIf) and the nutritional balance indexes in SSE (NBIs) were highly significant (p < 0.001) when DRIS methodologies were applied to the “Validation” database. These results validate the use of DRIS norms in the prediction of nutritional imbalances in Rosa spp. [31,32,52]. Since the “Validation” database comprises data from 119 samples taken throughout the study area, including a wide variety of soils, standards can be used with a high probability of success, independent of the crop location within the Bogotá plateau.

**Fig 1:**
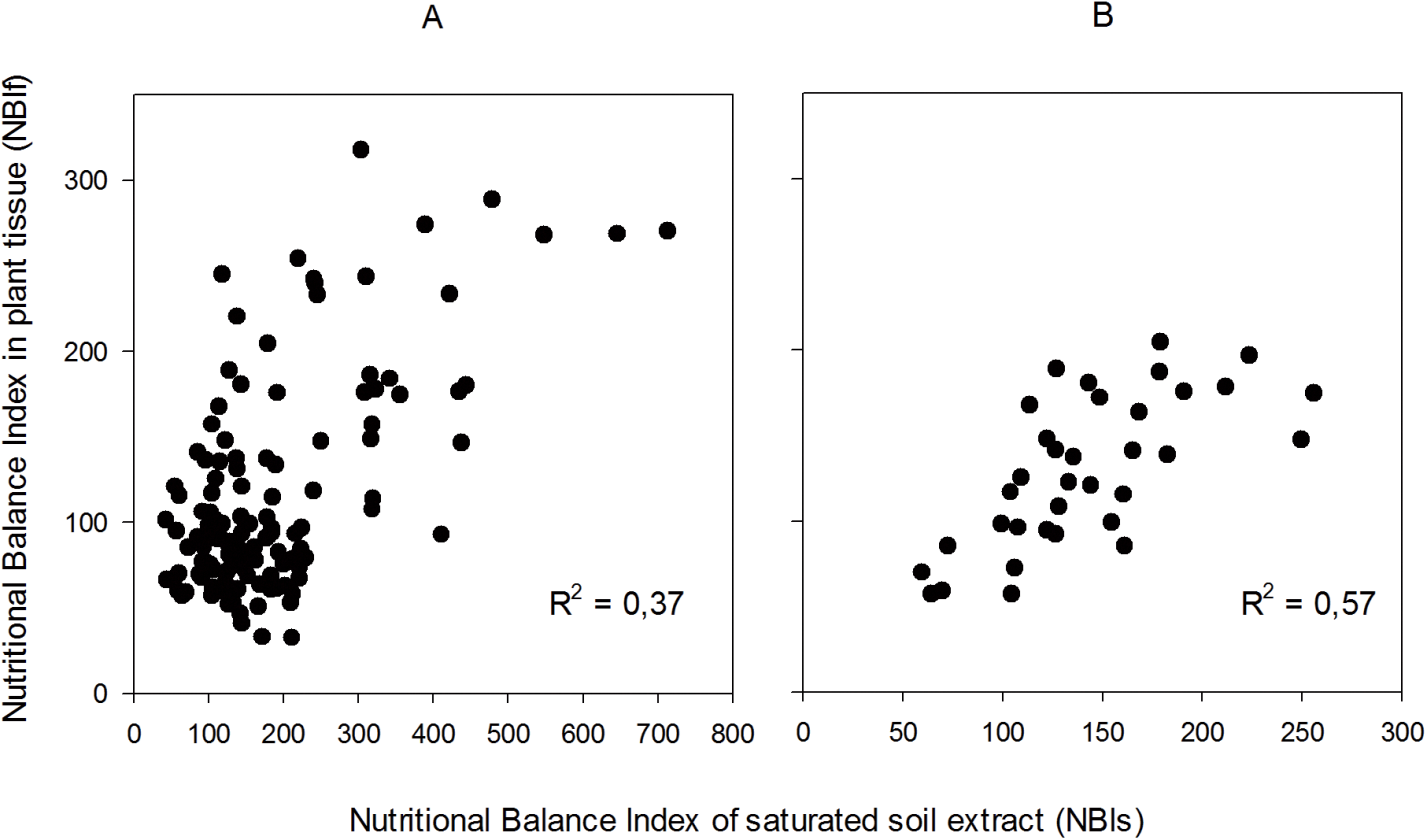
Correlations between NBIf (plant tissue) and NBIs (SSE) index. **pH restriction.** (A) pH range: 4.4 and 7.0. p<0.001; n = 124. (B) pH range 5.8 and 6.2. p<0.001; n = 35.

Because the pH values are not included within the DRIS norms and undoubtedly affect the nutritional status of a crop [11,46], the “Validation” database was limited to a range of pH (>5.8 and <6.2), considered as the most suitable for roses [34,42,44,46]. With this limitation, the value of the correlation coefficient R^2^ was improved from 0.37 to 0.57 (Fig 1), indicating the effective influence of pH on the nutritional balance of the crop.

These results suggest that the nutritional balance of rose tissues is closely related to the pH of soil [10,11]. In the same way, these results limit the use of DRIS norms to soils with a pH in the range of 5.8–6.2 observed in this study. In addition, this observation supports the need to include pH in soil nutritional diagnosis methods to improve an integrated diagnosis of the crop via DRIS [40]. The first step to obtain a nutritional balance in the plant/crop that will in turn lead to high yields is to control soil pH values.

**Fig 2:**
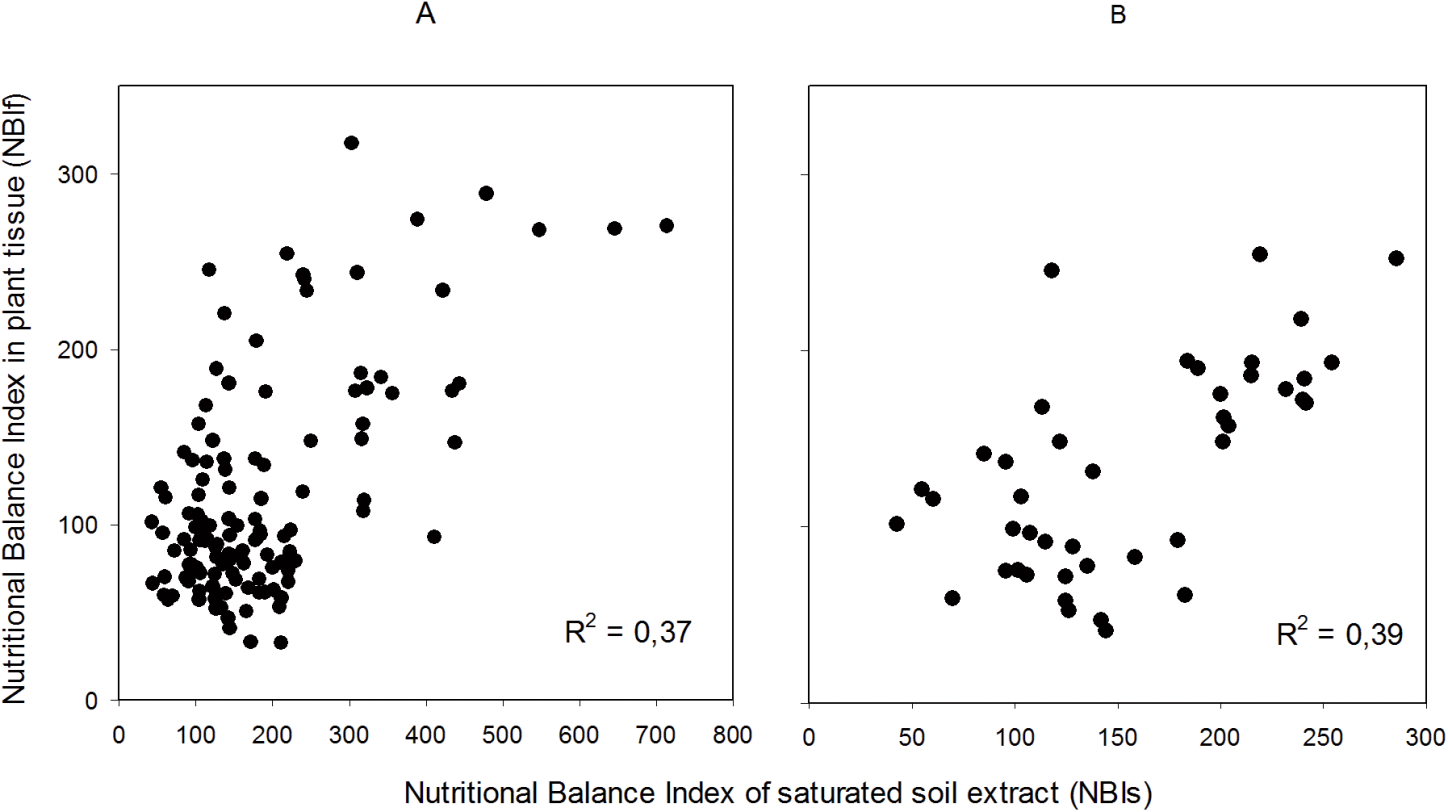
Correlations between NBIf (plant tissue) and NBIs (SSE) index. **Electric Conductivity (EC) restriction.** (A) EC range: 1.1 and 5.8 dS m-1. p<0.001 n = 124. (B) EC range 1.8 and 2.5. p<0.001 n = 35.

For similar reasons, the “Validation” database was limited in the total content of soluble salts measured indirectly by the EC of the SSE. The selected range was >1.8 and <2.5 dS m^-1^, values where rose plants do not present physiological disorders related to salinity [35,41]. This generated a small increase in the value of the correlation coefficient R^2^ from 0.37 to 0.39 (Fig 2). This small improvement indicates that although EC can have some impact on the result of diagnosis, its effects are considered within the DRIS indices of each element.

### CND norms

#### Separation of populations

Applying the Cate Nelson method (cited by [53,54]), to the “Source” database will get cumulative proportion of variance functions and the critical value for CNDr^2^. This critical value can be used to separate the population with a high nutritional balance in leaf tissues from the low-nutritional balance population. In this case, the CNDr^2^ separation value between the two populations would be 9.9 corresponding to N-NH_4_ (lowest value of all functions of the proportion of variance; (S3 Table). However, the results obtained by Franco et al. [1] indicate that CND r^2^ values greater than 7.4 in rose tissues have a high probability of belonging to the low-yielding population. This population will be able to provide a suitable diagnosis for describing the nutritional balance in the soil that may lead to a proper nutritional balance in plant. This method of separation, although not strictly limited to the original method proposed by Khiari [53,54], is fully consistent with the principles of integrated diagnostic systems that require a truly exceptional reference population. This separation allows differentiate the elements, which generated significant effects on the nutritional balance of populations (**Table 2**). Clarifying analysis, the effort can focus on those elements that have greater impact on nutritional balance and presumably on productivity.

**Table 2:** Basic statistics in populations with high and low nutritional balance, and results of the *F*-test and *t-*test applied to the calculation of CND norms of saturated soil extract analysis from rose crops. (Foliar CND r^2^ <7.4 on high nutritional balance population).

	*High Nutritional Balance Population*				*Low Nutritional Balance Population*				*F*-test (*S*_h_ < *S*_l_)	*t*-test (μ_h_ ≠ μ_l_)
	Mean (μ_h_)	CV%	Median	*S*_h_	Mean (μ_l_)	CV %	Median	*S*_l_		
***CND r***^***2***^ ***F***	6.0	23	6.0	1.4	19.9	42	18.8	8.4	6*	0*
***EC***	2.6	32	2.4	0.84	2.8	39	2.7	1.08	1,29	0,04*
***pH***	6.1	7	6.1	0.45	6.0	9	6.0	0.54	1,2	0,5
***N-NO***_***3***_	169.8	39	156.8	65.8	177.4	49	173.5	86.6	1,32	0,07
***N-NH***_***4***_	9.7	63	9.1	6	8.4	65	8.1	5	0,83	0,02*
***P***	3.4	55	3.4	1.9	5.8	143	5.5	8.3	4,37*	0*
***K***	106.5	41	102.9	43	129.6	52	126.2	68	1,58	0,08
***Ca***	232.9	37	226.1	87	250.4	47	248.8	118	1,36	0,04*
***Mg***	98.5	34	96.5	34	109.0	53	108.0	58	1,71	0,03*
***S-SO***_***4***_	188.1	39	170.3	73	216.1	53	219.0	113	1,55	0,02*
***Cl***	111.6	58	110.3	65	91.2	75	88.5	69	1,06	0,1
***Na***	114.6	60	108.0	69	97.5	69	90.5	67	0,97	0,2
***Fe***	0.69	46	0.6	0.32	1.02	76	0.9	0.77	2,41*	0*
***Cu***	0.11	43	0.1	0.046	0.12	78	0.1	0.091	1,98*	0,02*
***Mn***	0.32	32	0.2	0.103	0.23	348	0.4	0.812	7,88*	0*
***Zn***	0.23	49	0.2	0.115	0.26	134	0.2	0.352	3,06*	0,02*
***B***	0.96	28	1.0	0.27	1.10	38	1.0	0.42	1,56	0,1

CND r^2^ F: Foliar CND nutritional balance index. EC: (dS m^-1^). Element units in mg L^-1^. CV (%): Coefficient of Variation. S_h_ and μ_h_: Variance and Means of the high nutritional balance population. S_l_ and μ_l_: Variance and mean of the low nutritional balance population.

Values indicated * are statistically significant at α = 0.05.

#### Ratio significance and CND norms

Basic statistics of populations obtained with this separation method are shown in **Table 2**. There are significant differences between the two populations on the variance and many of the mean values. This confirms that the selected population of high CNDf is markedly different from the low CNDf group and generates reliable diagnostic CND norms for soil SSE analyses (CNDs).

The EC presents significant differences in mean between the two populations expressing lower results in the population of high CND*s* (**Table 2**). Moreover, it was found that most elements (except: N-NH_4_, Cl, Na and Mn) have lower average concentration values in the population of high CND*f* than in the population of low CND*f*. As previously discussed in the DRIS norms section, high concentrations of elements in the soils do not necessarily indicate optimum flower crop yields.

Phosphorus, Fe, Cu and Zn, show significant differences both on variance and in the mean, highlighting that the high-performance population values are lower in all cases. This suggests an over-fertilization with these elements. These data is highlighted when compared with the mean values referred by Cabrera [35] or those usually applied in the Bogotá plateau region (S4 Table). The application of high quantities of the mentioned elements, have been historically justified, based on the high content of organic matter and volcanic ash that exist in the Bogotá plateau soils (**Table 1**). The concentrations of P, Fe, Zn and Cu in fertigation solutions should be checked. The over-fertilization with these elements generates imbalances in SSE, which also reflect imbalances in the nutrient status of rose plants. These imbalances are likely to be the cause of reduced flower productivities and undoubtedly increases in environmental pollution.

Conversely, Mn values are significantly higher in high-performance population. This result is particularly significant considering the low concentration of extractable Mn in soils (Table 1). These data, coupled with low concentrations of Fe, Cu and Zn in SSE, display a critical situation for this element in the rose production system.

Focusing on nutritional balance, reductions in current fertigation programs for the above-mentioned elements, will probably improve the availability of manganese in the soil. Nevertheless, special attention should be paid to Mn concentrations in the soil-plant system, when designing fertigation solutions.

The CNDs norms, from analysis of saturated soil extracts, were calculated (**Table 3**). Rose crops with values of CND*f* <7.4 are considered to have a high probability of belonging to a high yield (>137 flowers/m^2^/yr.) population [1].

**Table 3:** CND norms from analysis of saturated soil extracts (SSE) for *Rose sp*. growing in the Bogotá Plateau, Colombia.

	Norm (V*)	SD
**N-NO3**	2,60	0,27
**N-NH4**	-0,41	0,14
**P**	-1,43	0,64
**K**	2,14	0,30
**Ca**	2,93	0,26
**Mg**	2,07	0,26
**S-SO4**	2,71	0,32
**Cl**	2,07	0,61
**Na**	2,13	0,48
**Fe**	-2,92	0,50
**Cu**	-4,91	0,83
**Mn**	-4,36	1,02
**Zn**	-4,03	0,39
**B**	-2,53	0,22
**R**	3,94	0,25

Norm (V*): CND Norms for each element (units in mg L^-1^). SD: Standard deviation

The coefficients of variation (CV) (**Table 2**) are lower than those presented for DRIS norms (S2 Table). This is because CND norms are obtained from normalized values in a singular relationship for each individual element, as opposed to the binary ratios implemented for the DRIS system [55].

Only 4.6% of SSE in the “Source” database was considered within the high yield population. Thus, there is a probability (*p* = 0.954) that an individual observation is underperforming. The CND r^2^ values were distributed as a *Chi*-square function likelihood with 16 degrees of freedom. In theory, all CNDs >7.8 have a high probability of being in the low yield population.

#### Theoretical validation of CND norms

The theoretical validation of the CND norms was conducted applying CND methodologies to the “Validation” database. Highly significant correlations were obtained (p < 0.01), with a R^2^ of 0.57) between CNDf and CNDs. This result validates the use of diagnostic CND norms in the prediction of nutritional imbalances in rose crops [13,32,52].

The R^2^ correlation coefficients obtained for the “Validation” database between CND*f* and CND*s* are much higher than those found with the DRIS methodology for equivalent NBI*f* and NBI*s*. This aspect can be based on the methodology proposed by Parent et al. [30], which does not determine the concentration of the element in the solution, but their participation percentage in the total soluble salts. Thus, the participation percentage of each element is related to the participation of the other elements in the total soluble salts in solution. These relationships are more closely related to the chemical equilibrium in solutions (which probably are higher using isometric log ratios) [39,56] and therefore, with the availability of nutrients for the plants [8–11].

**Fig 3:**
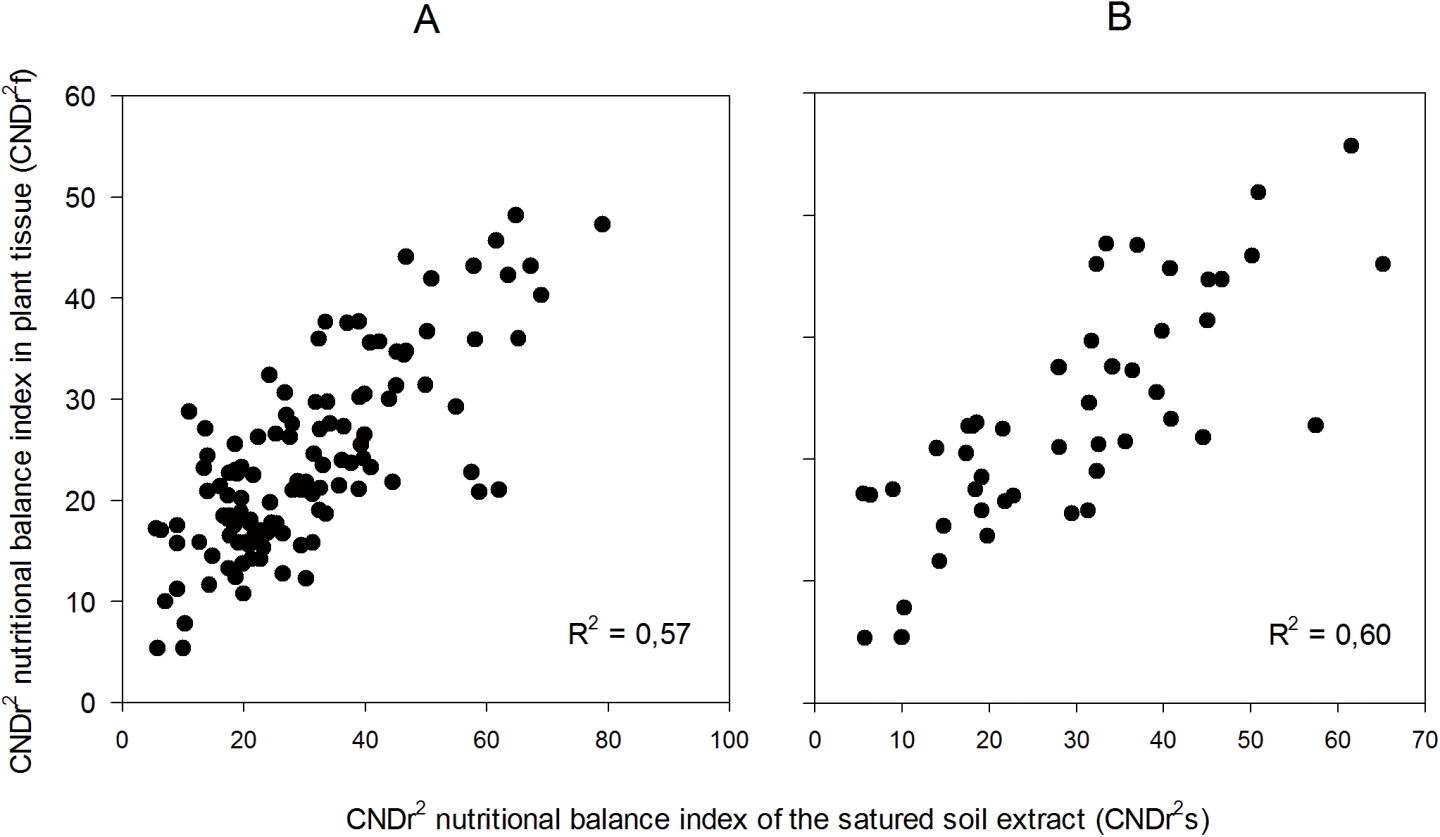
Correlations between CND*f* (plant tissue) and 345 CNDs (SSE) index. **pH restriction.** pH range: 4.4 and 7.0. p<0.001; n = 124. (B) pH range 5.8 and 6.2. p<0.001; n = 35.

**Fig 4:**
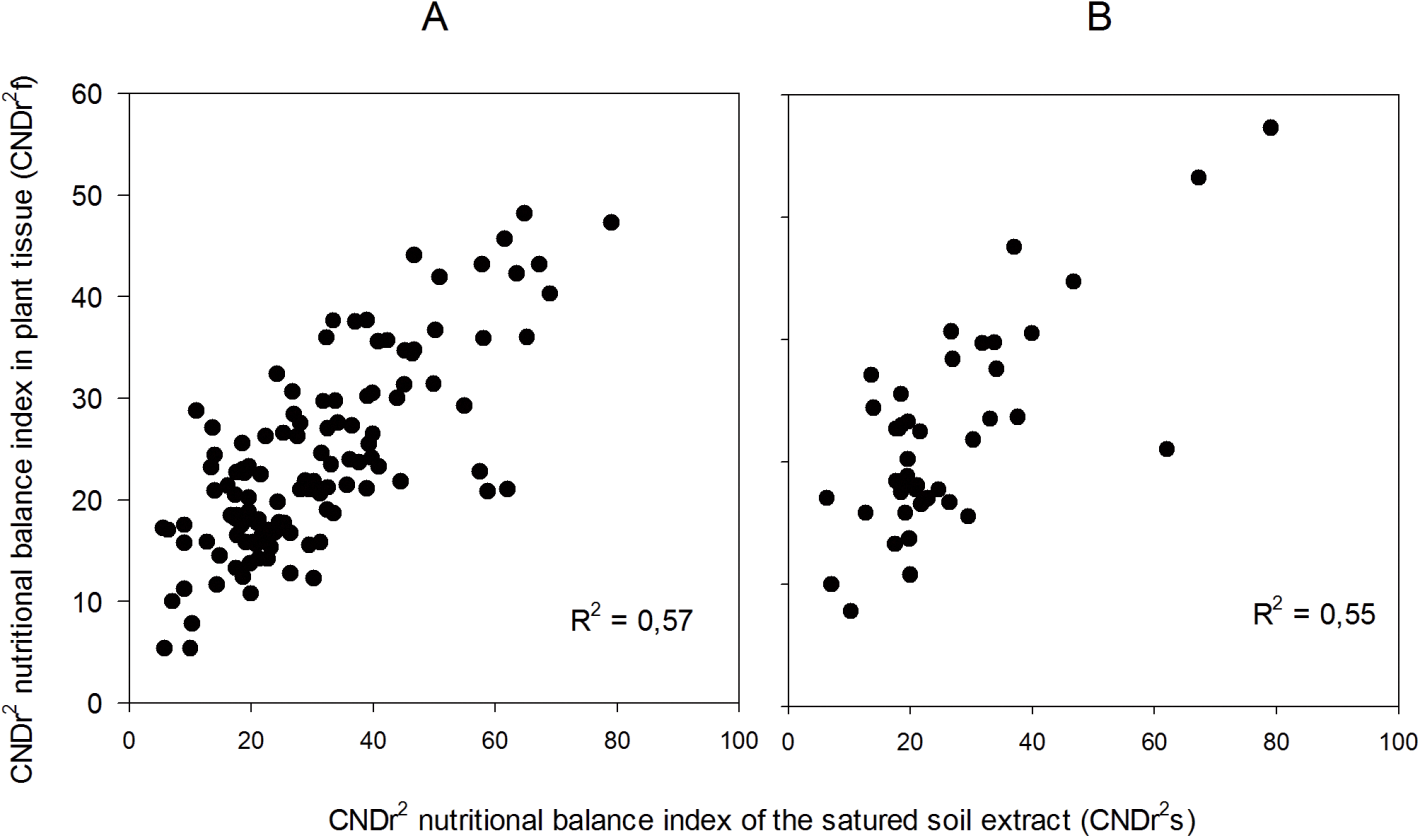
Correlations between CND*f* (plant tissue) and CND*s* (SSE) index. **Electric Conductivity (EC) restriction.** (A) EC range: 1.1 and 5.8 dS m^-1^. p<0.001 n = 124. (B) EC range 1.8 and 2.5. p<0.001 n = 35.

As already mentioned in the DRIS norms section, pH values were not included within the CND diagnosis norms, despite the fact that pH strongly affects both the availability of elements and the nutritional status of crops [11,46,57]. For this reason, the “Validation” database was limited to the same pH range (>5.8 and <6.2), which contributed to a slight increase in the value of R^2^ (from 0.57 to 0.60; Fig 3). In both methodologies, the correlation coefficients were closer when the pH values in SSE were limited to the optimal range as reported in previous studies. These results suggest that variability in nutrient balance is closely related to SSE pH. Similarly, the database was limited to the EC of the SSE (>1.8 and <2.5 dS m^-1^), in this case obtaining a reduction in the value of R^2^ (from 0.57 to 0.55; Fig 4), which indicates that the effect of EC is already contemplated in the CND diagnosis. CND method can provide unbiased indices of nutrient balance in soils and plant tissues discarding random information and retains only significant factors [22,56,58].

## Conclusions

Considering the inter-relationship between soil and nutritional balance in plants, the results obtained in this study indicate that the CND method, compared to the DRIS method, defines closer relationships between soil solution and leaf tissue data Thus, the CND method is recommendable for nutritional diagnosis on cut rose crops, including instances when the pH and EC values in SSE are variable.

The use of the CND method can be a great utility tool to guide nutrient management in fertigated rose production systems, avoiding over-fertilization issues (and its associated pollution) in the Bogotá plateau and other flower growing regions of Colombia.

The main recommendations to increase and improve rose crop flower yields would be: continuously check pH values of SSE, reduce the amounts of P, Fe, Zn and Cu in fertigation solutions and analyze the situation of Mn in the soil-plant system.

## Supporting information

S1 Table Basic statistics in rose plant populations with high and low foliar nutritional balance indexes (NBI*f*) and results of *F*- and *t-*tests applied to the calculation of DRIS norms of saturated soil extracts from rose crops in the Bogota Plateu (Colombia). NBI*f*: Nutritional Balance Index of Leaf. EC: electrical conductivity (dS m^-1^), Element Units in mg L^-1^. CV (%): Coefficient of Variation. S_h_ and μ_h_ = Variance and means of the high nutritional balance population. *S*_l_ and μ_l_ = Variance and means of low nutritional balance population. Values indicated * are statistically significant (p = 0.05); 477 freedom degree for high nutritional balance population and 81 for low balance population.
(DOCX)

S2 Table Ratios and DRIS norms of saturated soil extracts from a rose population with a DRIS foliar tissue < 60. CV: Coefficient Variation. S_h_. S_l_ and μ_h_. μ_l_ = variance and means of high and low nutritional balance populations respectively. Element values in mg L^-1^. * Significant Values α = 0.05.
(DOCX)

S3 Table Functions of accumulated variance proportions for each element Fc(Vx), where “CND r^2^ F” is the CND nutritional balance index for foliar tissue which is the criterion for the separation of populations. (DOCX)

S4 Table Chemical composition of fertigation solutions used to fertilize greenhouse rose crops. * (Cabrera 2006). **Typical average values for roses crops grown in Colombia.
(DOCX)
