# Supplementary material for: Determination of diagnostic standards on saturated soil extracts for cut roses grown in greenhouses
Source: PLoS One. 2017 May 25;12(5):e0178500. doi: 10.1371/journal.pone.0178500 (PMC5444843; doi:10.1371/journal.pone.0178500)
Supplement: S1 Table — NBIf: Nutritional Balance Index of Leaf. EC: electrical conductivity (dS m-1), Element Units in mg L-1. CV (%): Coefficient of Variation. Sh and μh = Variance and means of the high nutritional balance population. Sl and μl = Variance and means of low nutritional balance population. Values indicated * are statistically significant (p = 0.05); 477 freedom degree for high nutritional balance population and 81 for low balance population. (DOCX) [file pone.0178500.s001.docx]

**S1 Table. Basic statistics in rose plant populations with high and low foliar nutritional balance indexes (NBI*f*) and results of *F*- and *t-*tests applied to the calculation of DRIS norms of saturated soil extracts from rose crops in the Bogota Plateu (Colombia).**

|  | *Population with High Nutritional Balance* | | | | *Population with Low Nutritional Balance* | | | | *F*-test  (*S*_h_ < *S*_l_) | *t-*test  (µ_h_ ≠ µ_l_) |
| --- | --- | --- | --- | --- | --- | --- | --- | --- | --- | --- |
|  | Mean | CV % | Median | *S*_h_ | Mean | CV % | Median | *S*_l_ |  |  |
| NBI*f* | 49.3 | 16.2 | 51.1 | 63.6 | 100.0 | 34.2 | 98.5 | 1170.6 | 18,41* | 0* |
| EC | 2.6 | 27.8 | 2.6 | 0.52 | 2.8 | 39.5 | 2.6 | 1.22 | 2,35* | 0,04* |
| pH | 5.9 | 8.5 | 5.9 | 0.25 | 6.0 | 8.9 | 6.0 | 0.29 | 1,16 | 0,17 |
| N_NH_4_ | 7.6 | 56.4 | 7.4 | 18.3 | 7.7 | 65.7 | 7.1 | 32.6 | 1,78* | 0,07 |
| N_NO_3_ | 164.4 | 33.1 | 162.3 | 2965 | 179.2 | 49.7 | 177.4 | 7934 | 2,68* | 0,02* |
| P | 4.2 | 85.4 | 3.3 | 12.7 | 5.8 | 142.8 | 5.6 | 69.4 | 5,46* | 0* |
| K | 115.5 | 44.8 | 113.0 | 2681 | 130.7 | 52.9 | 125.4 | 4768 | 1,78* | 0,03* |
| Ca | 232.6 | 37.0 | 227.4 | 7406 | 251.1 | 47.4 | 247.2 | 14179 | 1,91* | 0* |
| Mg | 98.3 | 35.0 | 97.3 | 1184 | 109.5 | 54.7 | 108.0 | 3585 | 3,03* | 0,02* |
| S | 196.9 | 35.6 | 192.9 | 4903 | 215.0 | 52.7 | 214.4 | 12819 | 2,61* | 0,05 |
| Cl | 97.1 | 62.6 | 97.8 | 3695 | 98.0 | 66.0 | 99.4 | 4779 | 1,29 | 0,55 |
| Na | 99.5 | 69.4 | 97.6 | 4759 | 98.4 | 70.2 | 96.3 | 4769 | 1 | 0,75 |
| Fe | 0.95 | 70.8 | 0.94 | 0.45 | 1.01 | 76.7 | 0.92 | 0.60 | 1,33 | 0,01* |
| Cu | 0.12 | 53.6 | 0.11 | 0.004 | 0.12 | 83.9 | 0.10 | 0.011 | 2,75* | 0,02* |
| Mn | 0.31 | 94.0 | 0.27 | 0.085 | 0.25 | 164.6 | 0.21 | 0.537 | 6,32* | 0* |
| Zn | 0.28 | 48.2 | 0.27 | 0.018 | 0.27 | 135.5 | 0.25 | 0.138 | 7,67* | 0,11 |
| B | 1.08 | 37.5 | 1.04 | 0.17 | 1.10 | 38.3 | 1.04 | 0.18 | 1,06 | 0,83 |

NBI*f*: Nutritional Balance Index of Leaf. EC: electrical conductivity (dS m^-1^), Element Units in mg L^-1^.

CV (%): Coefficient of Variation. S_h_ and µ_h_= Variance and means of the high nutritional balance population. *S*_l_ and µ_l_ = Variance and means of low nutritional balance population. Values indicated * are statistically significant (p = 0.05); 477 freedom degree for high nutritional balance population and 81 for low balance population.
