# Supplementary material for: Determination of diagnostic standards on saturated soil extracts for cut roses grown in greenhouses
Source: PLoS One. 2017 May 25;12(5):e0178500. doi: 10.1371/journal.pone.0178500 (PMC5444843; doi:10.1371/journal.pone.0178500)
Supplement: S2 Table — CV: Coefficient Variation. Sh. Sl and μh. μl = variance and means of high and low nutritional balance populations respectively. Element values in mg L-1. * Significant Values α = 0.05. (DOCX) [file pone.0178500.s002.docx]

**S2 Table. Ratios and DRIS norms of saturated soil extracts from a rose population with a DRIS foliar tissue < 60.**

| **Ratio** | **Norm** | **CV (%)** | ***F* test** | ***t* test** | **Ratio** | **Norm** | **CV (%)** | ***F* test** | ***t* test** | **Ratio** | **Norm** | **CV (%)** | ***F* test** | ***t* test** |
| --- | --- | --- | --- | --- | --- | --- | --- | --- | --- | --- | --- | --- | --- | --- |
| N-NH_4_/N-NO_3_ | 0.05 | 73.78 | 1.53 * | 0.06 | P/100Fe | 0.09 | 185.95 | 3.02 * | 0.04 * | 100Zn/Mg | 0.30 | 51.67 | 6,66 * | 0,09 |
| N-NH_4_/P | 4.33 | 149.76 | 7.38 * | 0.01 * | 100Cu/P | 7.57 | 193.42 | 4.83 * | 0.62 | Mg/100B | 0.98 | 43.80 | 1,95 * | 0,83 |
| N-NH_4_/K | 0.08 | 82.20 | 1.82 * | 0.35 | P/100Mn | 0.24 | 115.49 | 5.83 * | 0 * | Cl/S | 0.54 | 65.88 | 2,89 * | 0,90 |
| N-NH_4_/Ca | 0.04 | 73.10 | 1.28 | 0.07 | P/100Zn | 0.19 | 118.76 | 3.73 * | 0 * | Na/S | 0.52 | 55.68 | 1,88 * | 0,81 |
| N-NH_4_/Mg | 0.09 | 68.84 | 1.58 * | 0.03 * | P/100B | 0.04 | 79.36 | 4.19 * | 0 * | S/100Fe | 3.45 | 95.96 | 2,01 * | 0,52 |
| N-NH_4_/Cl | 0.17 | 181.45 | 4.06 * | 0.02 * | K/Ca | 0.54 | 45.11 | 1.67 * | 0.04 * | 100Cu/S | 0.06 | 49.48 | 2,91 * | 0,06 |
| N-NH_4_/Na | 0.11 | 94.99 | 2.53 * | 0.09 | Mg/K | 0.94 | 42.87 | 1.53 * | 0.46 | 100Mn/S | 0.16 | 89.71 | 12,85 * | 0 * |
| N-NH_4_/100Fe | 0.14 | 100.15 | 2.52 * | 0.38 | K/S | 0.65 | 51.13 | 2.48 * | 0.01 * | 100Zn/S | 0.15 | 47.68 | 4,35 * | 0,10 |
| 100Cu/N-NH_4_ | 2.27 | 85.22 | 1.5 * | 0.35 | K/Cl | 2.28 | 114.68 | 1.4 * | 0.09 | 100B/S | 0.61 | 45.95 | 3,59 * | 0,04 * |
| N-NH_4_/100Mn | 0.45 | 88.78 | 11.7 * | 0 * | K/Na | 1.83 | 83.94 | 0.97 | 0.55 | Na/Cl | 1.59 | 155.67 | 2,18 * | 0,21 |
| 100Zn/N-NH_4_ | 5.47 | 90.01 | 3.74 * | 0.30 | K/100Fe | 2.10 | 122.89 | 1.86 * | 0.64 | 100Fe/Cl | 2.91 | 173.32 | 2,19 * | 0,06 |
| N-NH_4_/100B | 0.08 | 71.90 | 1.26 | 0.13 | 100Cu/K | 0.11 | 58.62 | 3.09 * | 0.57 | 100Cu/Cl | 0.26 | 179.90 | 1,49 * | 0,27 |
| P/N-NO_3_ | 0.03 | 106.40 | 14.05 * | 0 * | 100Mn/K | 0.29 | 107.64 | 7.49 * | 0 * | 100Mn/Cl | 0.74 | 209.60 | 9,16 * | 0 * |
| K/N-NO_3_ | 0.76 | 48.61 | 2.1 * | 0.07 | 100Zn/K | 0.27 | 56.07 | 3.81 * | 0.68 | 100Zn/Cl | 0.62 | 148.63 | 1,87 * | 0,03 * |
| Ca/N-NO_3_ | 1.47 | 35.05 | 3.64 * | 0.77 | K/100B | 1.11 | 36.12 | 1.62 * | 0.15 | 100B/Cl | 2.19 | 134.92 | 2,27 * | 0,05 * |
| Mg/N-NO_3_ | 0.63 | 39.56 | 3.49 * | 0.47 | Mg/Ca | 0.43 | 22.90 | 2.17 * | 0.42 | 100Fe/Na | 1.60 | 119.41 | 1,54 * | 0,14 |
| N-NO_3_/S | 0.90 | 38.60 | 5.02 * | 0.05 * | Ca/S | 1.22 | 24.70 | 3.57 * | 0.13 | 100Cu/Na | 0.18 | 108.15 | 4,31 * | 0,16 |
| Cl/N-NO_3_ | 0.62 | 63.37 | 2.49 * | 0.63 | Ca/Cl | 4.94 | 133.26 | 2.16 * | 0.15 | 100Mn/Na | 0.46 | 134.00 | 15,66 * | 0 * |
| Na/N-NO_3_ | 0.64 | 65.52 | 1.20 | 0.47 | Ca/Na | 3.35 | 72.84 | 1.7 * | 0.87 | 100Zn/Na | 0.41 | 87.83 | 4,41 * | 0,19 |
| N-NO_3_/100Fe | 2.88 | 90.14 | 2.82 * | 0.36 | Ca/100Fe | 3.88 | 81.47 | 2.31 * | 0.65 | Na/100B | 0.99 | 74.80 | 1,43 * | 0,59 |
| 100Cu/N-NO_3_ | 0.08 | 83.08 | 2.98 * | 0.26 | 100Cu/Ca | 0.06 | 69.79 | 5.32 * | 0.18 | Cu/Fe | 0.21 | 130.53 | 0,99 | 0,80 |
| 100Mn/N-NO_3_ | 0.20 | 102.72 | 27.92 * | 0.01 * | 100Mn/Ca | 0.14 | 89.83 | 20.09 * | 0 * | Mn/Fe | 0.38 | 72.75 | 12,85 * | 0,09 |
| 100Zn/N-NO_3_ | 0.18 | 61.77 | 4.09 * | 0.33 | 100Zn/Ca | 0.13 | 54.02 | 4.27 * | 0.26 | Zn/Fe | 0.40 | 65.46 | 6,52 * | 0,70 |
| 100B/N-NO_3_ | 0.72 | 47.71 | 10.89 * | 0.15 | Ca/100B | 2.38 | 54.04 | 3.19 * | 0.68 | B/Fe | 1.87 | 90.19 | 2,1 * | 0,78 |
| P/K | 0.04 | 96.16 | 6.24 * | 0.01 * | Mg/S | 0.52 | 29.25 | 4.09 * | 0.05 * | Mn/Cu | 3.96 | 140.29 | 3,62 * | 0,07 |
| P/Ca | 0.02 | 133.84 | 5.05 * | 0.02 * | Mg/Cl | 1.99 | 133.07 | 1.82 * | 0.16 | Cu/Zn | 0.49 | 71.28 | 2,22 * | 0,31 |
| P/Mg | 0.05 | 115.75 | 6.87 * | 0.01 * | Mg/Na | 1.39 | 68.84 | 1.20 | 0.67 | Cu/B | 0.11 | 47.73 | 4,42 * | 0,30 |
| P/S | 0.03 | 112.56 | 4.05 * | 0 * | Mg/100Fe | 1.68 | 84.47 | 5.91 * | 0.63 | Mn/Zn | 1.22 | 88.43 | 5,21 * | 0 * |
| Cl/P | 52.67 | 125.63 | 18.98 * | 0.01 * | 100Cu/Mg | 0.13 | 59.03 | 9.87 * | 0.1 | Mn/B | 0.31 | 109.52 | 8,05 * | 0 * |
| Na/P | 55.76 | 127.18 | 18.8 * | 0.04 * | 100Mn/Mg | 0.34 | 100.09 | 23.56 * | 0 * | Zn/B | 0.27 | 50.28 | 7,77 * | 0,25 |

CV: Coefficient Variation. S_h_. S_l_ and μ_h_. μ_l_  = variance and means of high and low nutritional balance populations respectively. Element values in mg L^-1^. * Significant Values α=0.05.
