# Supplementary material for: Determination of diagnostic standards on saturated soil extracts for cut roses grown in greenhouses
Source: PLoS One. 2017 May 25;12(5):e0178500. doi: 10.1371/journal.pone.0178500 (PMC5444843; doi:10.1371/journal.pone.0178500)
Supplement: S3 Table — (DOCX) [file pone.0178500.s003.docx]

**Table S3. Functions of accumulated variance proportions for each element Fc(Vx), where “CND r^2^ F” is the CND nutritional balance index for foliar tissue which is the criterion for the separation of populations.**

|  | **Function of Fc(Vx) for CND r^2^ F** | **Critical**  **“CND r2 F”** |
| --- | --- | --- |
| **N NO3** | Fc(VN_NO3) = -24.115 + (2.945 * CND r^2^ F) + (0.0802 * CND r2 F^2) - (0.00189 * CND r2 F^3) | 14.1 |
| **N NH4** | Fc(VN_NH4) = -24.040 + (3.787 * CND r^2^ F) + (0.0458 * CND r2 F^2) - (0.00154 * CND r2 F^3) | 9.9 |
| **P** | Fc(VP) = -21.969 + (2.171 * CND r^2^ F) + (0.129 * CND r2 F^2) - (0.00261 * CND r2 F^3) | 16.5 |
| **K** | Fc(VK) = -21.831 + (3.029 * CND r^2^ F) + (0.0614 * CND r2 F^2) - (0.00158 * CND r2 F^3) | 13.0 |
| **Ca** | Fc(VCa) = -22.205 + (2.621 * CND r2 F) + (0.102 * CND r2 F^2) - (0.00222 * CND r2 F^3) | 15.3 |
| **Mg** | Fc(VMg) = -22.030 + (2.658 * CND r2 F) + (0.0977 * CND r2 F^2) - (0.00215 * CND r2 F^3) | 15.1 |
| **S SO4** | Fc(VS-SO4) = -32.555 + (4.826 * CND r2 F) + (0.0908 * CND r2 F^2) - (0.00240 * CND r2 F^3) | 12.6 |
| **Cl** | Fc(VCl) = -28.739 + (3.792 * CND r2 F) + (0.0651 * CND r2 F^2) - (0.00191 * CND r2 F^3) | 11.4 |
| **Na** | Fc(VNa) = -26.497 + (3.551 * CND r2 F) + (0.0643 * CND r2 F^2) - (0.00181 * CND r2 F^3) | 11.8 |
| **Fe** | Fc(VFe) = -23.043 + (2.556 * CND r2 F) + (0.113 * CND r2 F^2) - (0.00245 * CND r2 F^3) | 15.4 |
| **Cu** | Fc(VCu) = -20.889 + (2.715 * CND r2 F) + (0.0769 * CND r2 F^2) - (0.00176 * CND r2 F^3) | 14.6 |
| **Mn** | Fc(VMn) = -18.106 + (2.023 * CND r2 F) + (0.117 * CND r2 F^2) - (0.00231 * CND r2 F^3) | 16.9 |
| **Zn** | Fc(VZn) = -29.292 + (4.156 * CND r2 F) + (0.0486 * CND r2 F^2) - (0.00153 * CND r2 F^3) | 10.6 |
| **B** | Fc(VB) = -22.452 + (2.680 * CND r2 F) + (0.0983 * CND r2 F^2) - (0.00217 * CND r2 F^3) | 15.1 |
| **R** | Fc(VR) = -29.073 + (3.785 * CND r2 F) + (0.0599 * CND r2 F^2) - (0.00181 * CND r2 F^3) | 11.0 |
