# Supplementary material for: Determination of diagnostic standards on saturated soil extracts for cut roses grown in greenhouses
Source: PLoS One. 2017 May 25;12(5):e0178500. doi: 10.1371/journal.pone.0178500 (PMC5444843; doi:10.1371/journal.pone.0178500)
Supplement: S4 Table — * (Cabrera 2006). **Typical average values for roses crops grown in Colombia. (DOCX) [file pone.0178500.s004.docx]

**Table S4. Chemical composition of fertigation solutions used to fertilize greenhouse rose crops.**

|  | N | P | K^+^ | Ca^2+^ | Mg^2+^ | Mn | Fe | Cu | Zn | B |
| --- | --- | --- | --- | --- | --- | --- | --- | --- | --- | --- |
|  | mg l^-1^ | | | | | | | | | |
| Cabrera* | 100-250 | 15-30 | 50-150 | 50-250 | 70-120 | 0.3-0.6 | 1-2 | 0.01-0.02 | 0.02-0.05 | 0.2-0.5 |
| Colombia** | 140-180 | 30-40 | 150-200 | 100-150 | 40-70 | 0.5-1 | 0.5-2 | 0.1-0.5 | 0.2-0.6 | 0.5-1 |

* (Cabrera 2006). **Typical average values for roses crops grown in Colombia
